# Overexpression of miR‐431‐5p Inhibits the Development of Hypertrophic Scars by Negatively Regulating the Expression of *ZEB1*

**DOI:** 10.1111/jocd.70533

**Published:** 2025-11-18

**Authors:** Yaqing Liu, Tonghao Yao, Xiaolin Miao, Suwen Zhang, Junjie Liao

**Affiliations:** ^1^ Medical Services Department Shanghai Changzheng Hospital Shanghai China; ^2^ Department of Plastic and Aesthetic Surgery The Second Affiliated Hospital of Harbin Medical University Harbin China; ^3^ Department of Dermatology and Venereology Tongde Hospital of Zhejiang Province Hangzhou China; ^4^ Department of Plastic Surgery Tongde Hospital of Zhejiang Province Hangzhou China; ^5^ Dermatology Department Wenzhou Ouhai District People's Hospital Wenzhou China

**Keywords:** HSFBs, hypertrophic scar, miR‐431‐5p, *ZEB1*

## Abstract

**Background:**

The exact mechanism underlying hypertrophic scar (HS) remains elusive at present.

**Aims:**

This study aimed to investigate the potential regulatory mechanism of microRNA‐431‐5p in HS.

**Patients/Methods:**

100 HS patients were recruited in this study. The relative expression of miR‐431‐5p, *ZEB1*, and *α‐SMA* was quantified using RT‐qPCR. Cell proliferation was assessed via the CCK‐8 assay. Cell migration was evaluated using the Transwell. Cell apoptosis was determined by flow cytometry. Inflammatory factors' levels were measured through ELISA (Enzyme‐Linked Immunosorbent Assay). The targeting regulatory relationship was confirmed by the dual‐luciferase assay.

**Results:**

miR‐431‐5p was significantly reduced in HS tissues. Overexpression of miR‐431‐5p markedly suppressed cell proliferation and migration, promoted apoptosis, downregulated the levels of *IL‐1β* (*Interleukin‐1β*) and *IL‐6* (*Interleukin‐6*), and inhibited *α‐SMA* expression. Moreover, *ZEB1* was highly expressed in HS tissues, and miR‐431‐5p negatively regulated *ZEB1*. Further investigation revealed that overexpression of *ZEB1* could partially reverse the regulatory effects of miR‐431‐5p on cellular behavior.

**Conclusions:**

The miR‐431‐5p/*ZEB1* axis was involved in the regulation of HS.

## 1 Introduction

Hypertrophic scar (HS) is a complex and tightly regulated pathophysiological process characterized by excessive fibroblast proliferation, abnormal cellular migration, persistent inflammation, and dysregulated extracellular matrix remodeling. It is primarily driven by mechanisms such as excessive collagen production, overexpression of *α‐SMA*, and excessive deposition of extracellular matrix components [1, 2, 3]. HS not only induces uncomfortable symptoms such as itching, pain, and erythema but may also lead to cosmetic disfigurement. In severe cases, it can result in functional impairments and restrict their participation in daily activities due to social stigma and functional limitations [4, 5]. Currently, the primary treatment modalities for HS include surgical excision, radiotherapy, intradermal corticosteroid injections, and laser therapy [5, 6]. However, the precise regulatory mechanisms underlying HS formation remain largely elusive at present.

MicroRNAs (miRNAs) play essential roles in regulating cell metabolism and organismal homeostasis [7]. Alterations in miRNA expression are strongly associated with organ fibrosis and skin fibrosis [8]. In recent years, extensive research has demonstrated that multiple miRNAs are implicated in the onset and progression of HS [9, 10]. Specifically, engineered exosomes delivering high‐level miR‐141–3p can effectively suppress the pathological behaviors of scar fibroblasts [11]. Additionally, aberrant expression of miR‐22 plays a pivotal role in the pathogenesis of HS [12]. Studies have revealed that miR‐431‐5p expression is downregulated in colon cancer [13]. In osteosarcoma cells, overexpression of miR‐431‐5p induces apoptosis and significantly inhibits cell proliferation and migration [14]. Moreover, miR‐431‐5p expression is closely linked to the production of inflammatory factors [15]. miR‐431‐5p may modulate the functions of immune cells, thereby affecting the microenvironment of HS tissues [16]. However, the precise regulatory mechanism of miR‐431‐5p in HS remains elusive. A study found that inhibiting *ZEB1* may offer novel therapeutic strategies for renal fibrosis [17]. Research demonstrates that *ZEB1* gene knockout suppresses HS formation both in vitro and in vivo in mice [18]. Furthermore, database analyses suggested that *ZEB1* was a target gene of miR‐141–3p. Based on these findings, we hypothesized that miR‐141–3p contributed to HS progression by targeting and regulating *ZEB1*.

The objective of this study was to further elucidate the potential regulatory mechanism of the miR‐431‐5p/*ZEB1* axis in HS by investigating cell proliferation, cell migration, cell apoptosis, the expression of inflammatory factors, and *α‐SMA* levels, thereby providing novel insights for the treatment of HS.

## 2 Materials and Methods

### 2.1 Research Participants

100 HS patients admitted to Tongde Hospital of Zhejiang Province between January 2022 and October 2024 were recruited as research participants, comprising 49 males and 51 females. The inclusion criteria were as follows: (1) HS patients were assessed using the Patient and Observer Scar Assessment Scale (POSAS); (2) patients were aged between 18 and 60 years; (3) they had a clear history of skin injury (e.g., surgery, burns) and developed HS at the injured site; (4) scar tissue had persisted for more than 3 to 6 months; (5) scars were significantly elevated above the normal skin, with red or purple coloration, a smooth or nodular surface, and accompanied by varying degrees of itching, pain, or tightness. The exclusion criteria were as follows: (1) presence of severe underlying diseases (e.g., cardiovascular diseases); (2) psychiatric illness or cognitive impairment; (3) recent receipt of treatments that could potentially affect scar status (e.g., laser therapy, local glucocorticoid injection, surgical resection); (4) women who were pregnant or lactating. Specimens for the experimental group were obtained from HS tissues, while those for the control group were derived from normal skin tissues within a 3‐cm radius around the HS.

The distribution of scars among the included patients was as follows: 12 cases on the face, 18 cases on the neck, 43 cases on the trunk, and 27 cases on the extremities. The causes of HS were distributed as follows: 43 cases resulted from burns, 21 cases from trauma, and 36 cases from surgery.

This study was approved by the Ethics Committee of Tongde Hospital of Zhejiang Province. Both patients and their family members provided written informed consent, and all procedures followed the Helsinki Declaration.

### 2.2 Cell Culture and Transfection

Hidradenitis Suppurativa Fibroblasts (HSFBs) were obtained from the ATCC. The cells were maintained in a DMEM medium supplemented with 10% fetal bovine serum (FBS) and incubated at 37°C under a 5% CO_2_ atmosphere. Following trypsinization, the cells were resuspended in PBS, and the cell density was adjusted to 2 × 10^3^ cells/mL to prepare a single‐cell suspension for subsequent experiments.

miR‐431‐5p mimic and oe‐*ZEB1* (a *ZEB1* overexpression vector constructed using the recombinant pcDNA3.1 plasmid) were synthesized by RiboBio Co. Ltd. HSFBs were randomly allocated into five groups: control, mimic‐NC, miR‐mimic, miR‐mimic + oe‐NC, and miR‐mimic + oe‐*ZEB1*. The concentration of miR‐431‐5p‐mimic was 50 nM. The control group consisted of untreated HSFBs. Following the manufacturer's instructions for Lipofectamine 2000 (Invitrogen, USA), the respective plasmids were transfected into HSFBs and cultured continuously for 48 h.

### 2.3 Relative Expressions of miR‐431‐5p, ZEB1, and α‐SMA Were Measured by RT‐qPCR

Total RNA was extracted using the TRIzol method. Subsequently, the RNA was reverse‐transcribed into cDNA. RT‐qPCR was then performed using the mirVana RT‐qPCR miR Detection Kit (Thermo Fisher Scientific Inc.) in combination with SYBR Green fluorescent dye (Roche, Germany). The obtained cDNA served as the template for the PCR reactions. U6 and GAPDH were used as internal reference genes for normalization. The primers were obtained from Genecopoeia (Guangzhou, China). The primers for RT‐qPCR were as follows: miR‐431‐5p: 5′‐TGT CTT GCA GGC CGT CAT G‐3′ (Forward) and 5′‐GCT GTC AAC GAT ACG CTA CCT A‐3′ (Reverse); ZEB1: 5′‐AGCAGTGAAAGAGAAGGGAATGC‐3′ (Forward) and 5′‐GGTCCTCCTCAGGTGCCTCAG‐3′ (Reverse); α‐SMA: 5′‐TACTGCCGAGCCTGAGAT‐3′ (Forward) and 5′‐GCTTCGTCGTATTCCTGTTT‐3′ (Reverse); U6: 5′‐CTCGCTTCGGCAGCACA‐3′(Forward) and 5′‐AACGCTTCACGAATTTGCGT‐3′(Reverse); GAPDH: 5′‐GAA GGT GAA GGT CGG AGT C‐3′ (Forward) and 5′‐GAA GAT GGT GAT GGG ATT TC‐3′ (Reverse). The relative expression of the target gene was calculated by the 2^−ΔΔCt^ method. The difference in Ct values (ΔCt) between the target gene and the reference gene was calculated for each sample to account for variations in reference gene expression. Then, the difference in ΔCt values between the experimental group and the control group (ΔΔCt) was computed to standardize intergroup differences. Finally, using the formula “relative expression = 2^−ΔΔCt^” to determine the fold change in expression of the target gene within the experimental group relative to that of the control group.

### 2.4 CKK‐8 Assay

The cell activity was assessed using the CCK‐8 method. Cells in the logarithmic growth phase were seeded into 96‐well plates at a density of 5 × 10^3^ cells per well. At 0, 24, 48, and 72 h post seeding, 10 μL of CCK‐8 reagent was added to each well. Following an additional incubation period of 4 h, the absorbance values were measured.

### 2.5 Migration Assay

The cell suspension was transferred into the apical chamber of the Transwell. Serum‐free medium was added to the upper chamber, while 600 μL of medium supplemented with 10% FBS was added to the lower chamber. The Transwell system was then incubated at 37°C for 24 h. Upon completion of the incubation period, the cells were fixed using 4% paraformaldehyde and stained with 0.1% crystal violet. After staining, the cells were gently rinsed five times. Finally, the migrated cells were examined and quantified.

### 2.6 Apoptosis Assay

The HSFBs were trypsinized and subsequently rinsed. Annexin V‐FITC and PI staining solutions were then added, followed by incubation at 37°C for 60 min. Flow cytometry was used to detect the cell apoptosis rate. The final number of apoptotic cells was estimated as the total percentage of early apoptotic cells (staining positive for Annexin V and negative for PI) and late apoptotic cells (staining positive for both Annexin V and PI). The cell apoptosis rate was calculated using the following formula: Cell apoptosis rate (%) = (Number of apoptotic cells/Total number of cells) × 100.

### 2.7 ELISA (Enzyme‐Linked Immunosorbent Assay)

The expression of *IL‐1β* (*Interleukin‐1β*) and *IL‐6* (*Interleukin‐6*) was quantified using an ELISA kit. Following the completion of transfections, the ELISA 96‐well plates for inflammatory cytokines were washed three times, each lasting 5 min. Subsequently, 100 μL of pre‐cooled medium was added, with three replicate wells established for each group, followed by overnight incubation at 4°C. Subsequently, the secondary antibody provided in the kit was added and incubated at room temperature for 30 min. Thereafter, the wells were washed three additional times with the wash buffer, each lasting 5 min, before proceeding with the color development reaction. Finally, the OD values were measured.

### 2.8 Dual‐Luciferase Reporter Assay

The wild‐type *ZEB1* (*ZEB1*‐WT) or its mutant form (*ZEB1*‐MUT) was cloned into the pGL3 reporter plasmid. Subsequently, miR mimics, miR inhibitors, or respective negative controls were co‐transfected with reporter plasmids containing the *ZEB1* (WT or MUT) or the empty vector pGL3 plasmid into HSFBs via Lipofectamine 2000.

### 2.9 Statistical Analysis

Data processing was performed using SPSS 21 statistical software and GraphPad Prism 9. Data normality was assessed using the Kolmogorov–Smirnov (K–S) test. For non‐normally distributed continuous variables, intergroup differences between two groups were evaluated via the Mann–Whitney *U* test. Independent‐samples t‐tests were performed for two‐group comparisons of normally distributed variables, whereas one‐way ANOVA was applied for multiple‐group comparisons. Additionally, correlation analysis was conducted using the Pearson correlation coefficient method. Each cell experiment was repeated three times. The threshold for statistical significance was set at *p* < 0.05.

## 3 Results

### 3.1 MiR‐431‐5p Was Downregulated in HS Tissues

**FIGURE 1:**
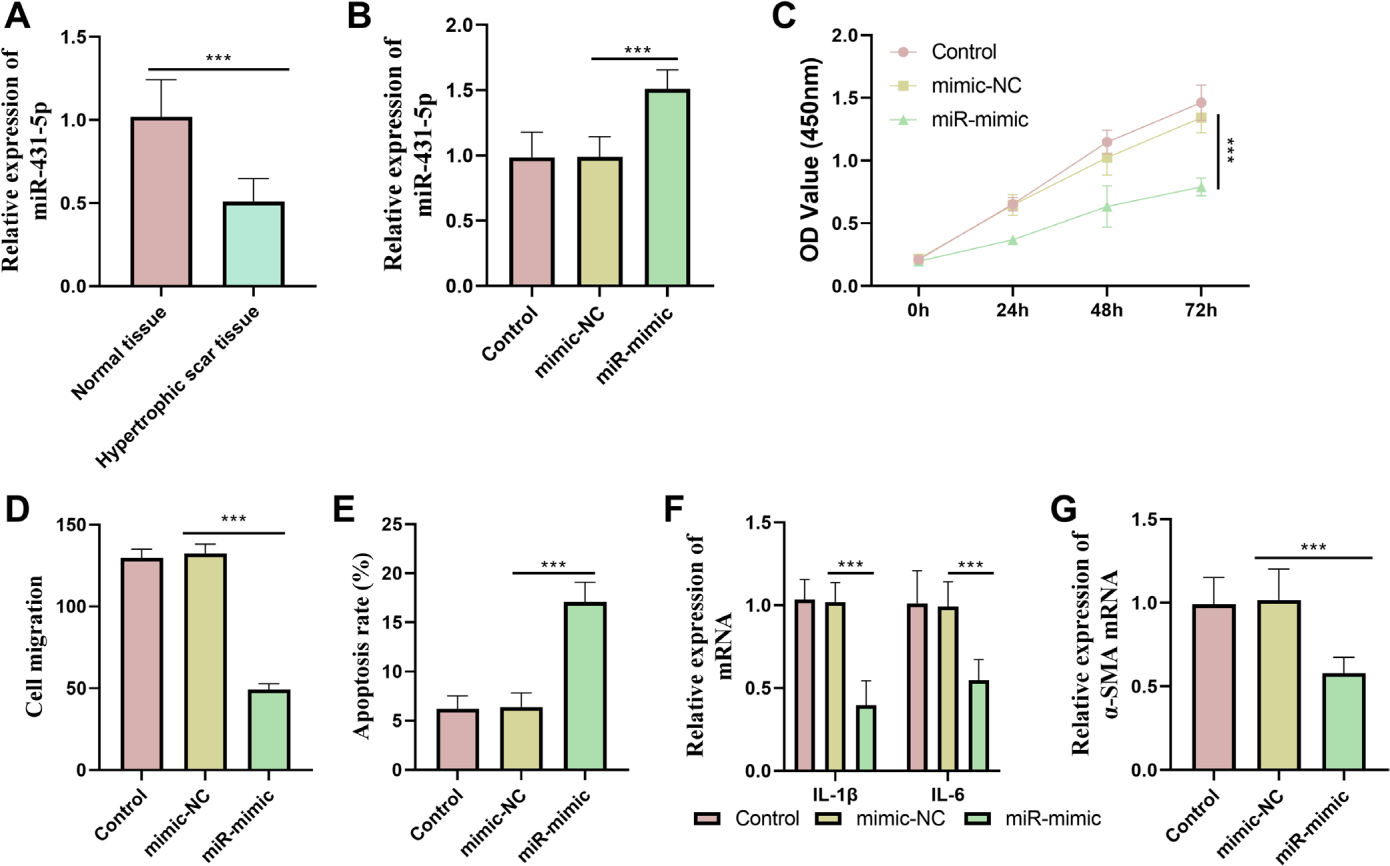
MiR‐431‐5p was downregulated in HS tissues. (A) Comparison of the relative expression of miR‐431‐5p in normal tissue and HS tissue. (B) Transfection of miR‐mimic could effectively increase the expression of miR‐431‐5p. (C, D) miR‐431‐5p inhibited cell proliferation and migration. (E) Overexpression of miR‐431‐5p promoted cell apoptosis. (F) Overexpression of miR‐431‐5p could reduce the expression of inflammatory factors. (G) Overexpression of miR‐431‐5p could inhibit the expression of *α‐SMA*. Non‐parametric test was used as statistical methods. ***p < 0.001.

MiR‐431‐5p was significantly downregulated in HS tissues (Figure 1A). Upon transfection with a miR‐431‐5p mimic, miR‐431‐5p was markedly upregulated (Figure 1B). Overexpression of miR‐431‐5p effectively suppressed cell proliferation and migration (Figure 1C,D), promoted apoptosis (Figure 1E), reduced the *IL‐1β* and *IL‐6* levels (Figure 1F), and inhibited the *α‐SMA* expression (Figure 1G).

### 3.2 The Targeting Relationship Between miR‐431‐5p and ZEB1

**FIGURE 2:**
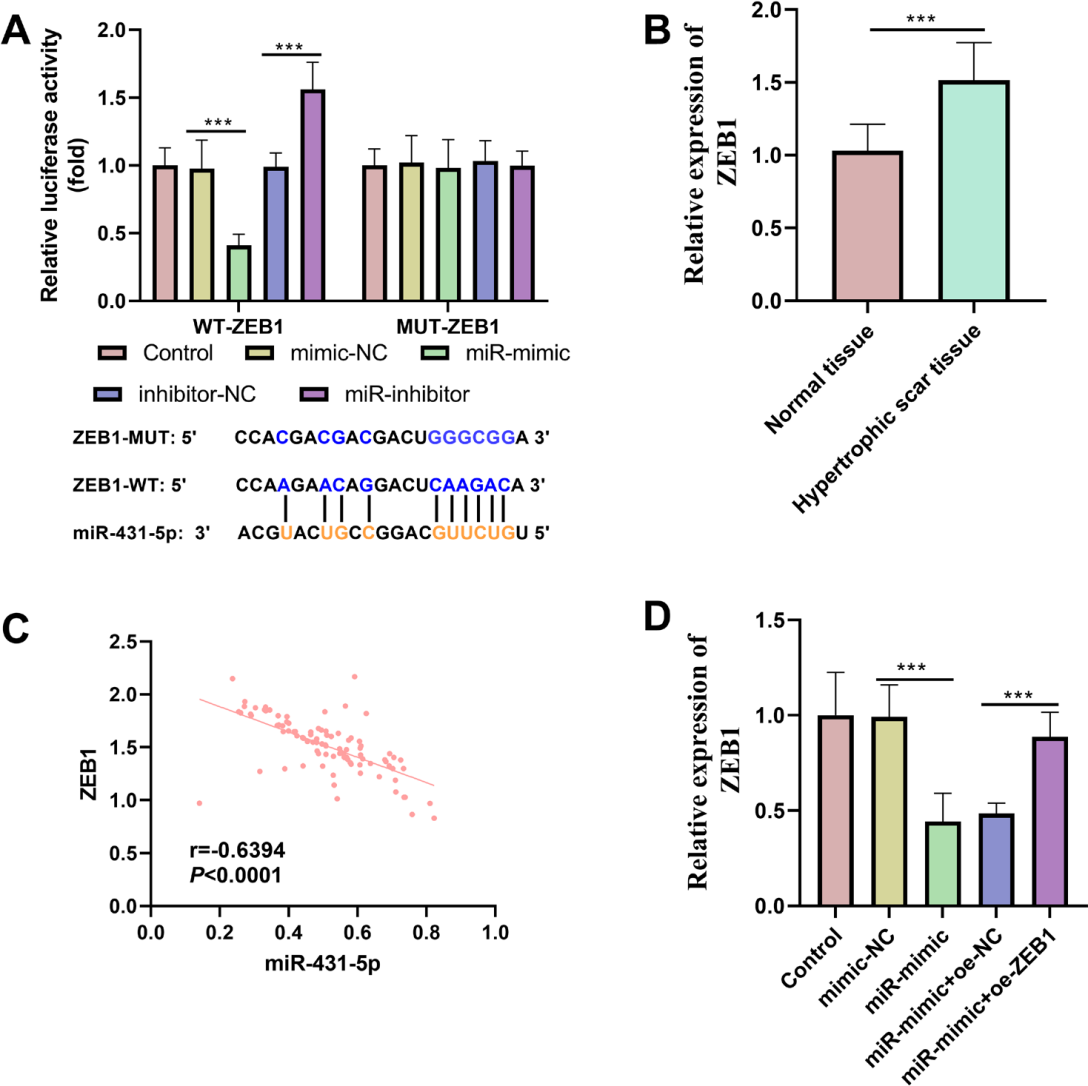
The targeting relationship between miR‐431‐5p and *ZEB1*. (A) The targeting relationship between miR‐431‐5p and *ZEB1* was verified by dual‐luciferase reporter gene assay. (B) Comparison of the relative expression of *ZEB1* in normal tissue and HS tissue. (C) The expression of miR‐431‐5p was significantly negatively correlated with *ZEB1*. (D) Relative expression of *ZEB1* in different treatment groups. Non‐parametric test and Pearson correlation coefficient method were used as statistical methods. ***p < 0.001.

In WT‐*ZEB1*, transfection with miR‐mimic markedly decreased luciferase activity, whereas transfection with miR‐inhibitor significantly enhanced it. Conversely, in MUT‐*ZEB1*, no significant alteration in luciferase activity was observed regardless of whether miR‐431‐5p was overexpressed or inhibited, further substantiating the targeted regulatory relationship between miR‐431‐5p and *ZEB1* (Figure 2A). Moreover, *ZEB1* in HS tissues was substantially elevated (Figure 2B). Correlation analysis revealed a significant negative correlation between miR‐431‐5p and *ZEB1* expression (Figure 2C). MiR‐431‐5p overexpression markedly reduced *ZEB1* expression, and co‐transfection with the oe‐*ZEB1* plasmid effectively restored its expression, thus reinforcing the conclusion that miR‐431‐5p suppressed *ZEB1* expression via targeted regulation (Figure 2D).

### 3.3 The Dynamic Interaction Relationship Between miR‐431‐5p and ZEB1

**FIGURE 3:**
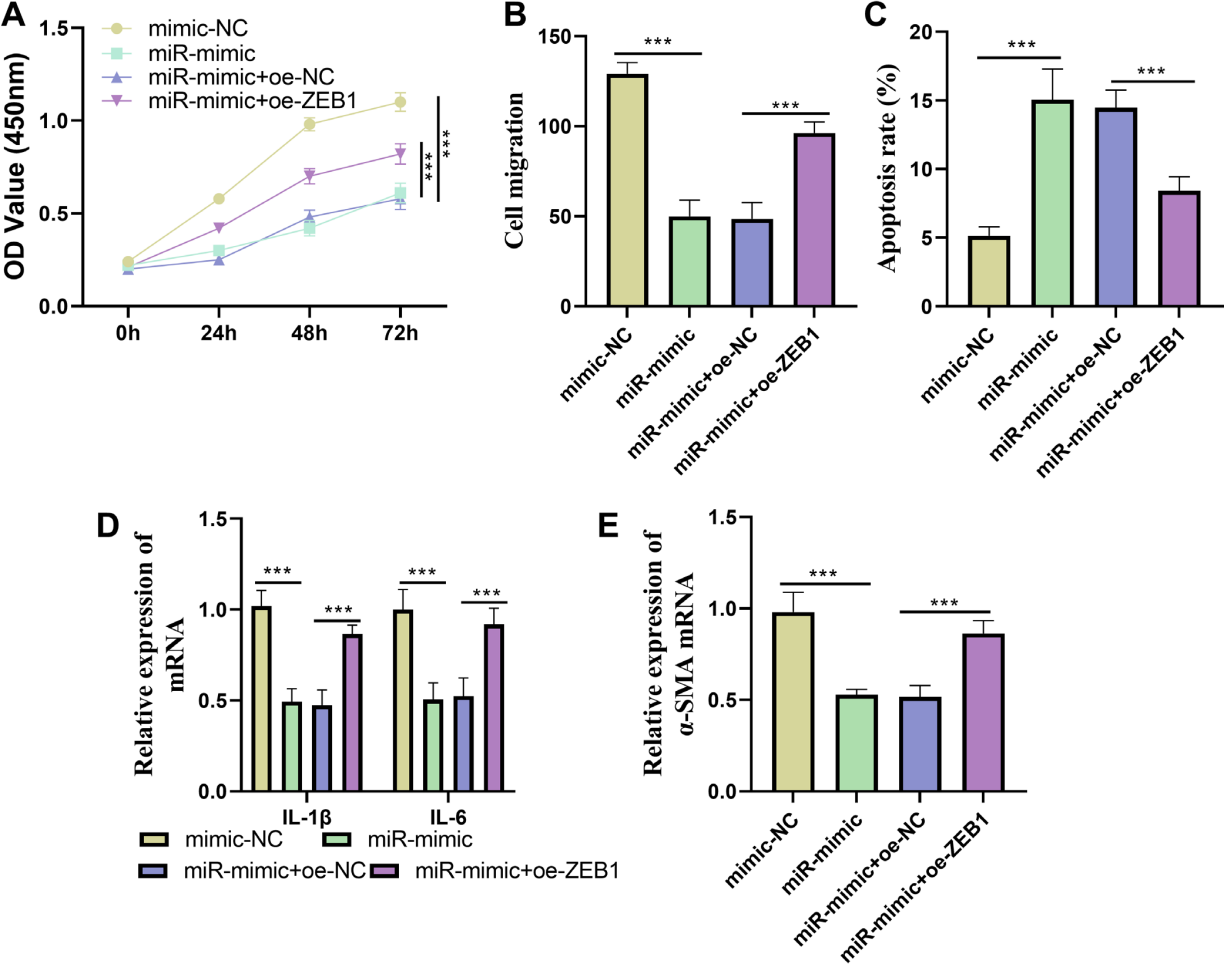
The dynamic interaction relationship between miR‐431‐5p and *ZEB1*. (A–C) Overexpression of *ZEB1* could partially reverse the inhibitory effect of miR‐431‐5p on cell proliferation and migration, as well as its promoting effect on apoptosis. (D) Overexpression of *ZEB1* could partially reverse the inhibition of miR‐431‐5p on the expression of inflammatory factors. (E) Overexpression of *ZEB1* could partially reverse the inhibitory effect of miR‐431‐5p on the *α‐SMA* expression. Non‐parametric tests were used as statistical methods. ***p < 0.001.

MiR‐431‐5p exerted its biological functions through the direct targeting and regulation of *ZEB1*. Functional experiment results demonstrated that the overexpression of *ZEB1* could partially reverse the inhibitory effects of miR‐431‐5p on cell proliferation and migration (Figure 3A,B), partially counteract the pro‐apoptotic effect of miR‐431‐5p (Figure 3C), and partially alleviate the suppressive effects of miR‐431‐5p on the expression of inflammatory factors and *α‐SMA* (Figure 3D,E). These findings further confirmed that miR‐431‐5p achieved its functional roles by directly regulating *ZEB1*.

## 4 Discussion

HS is characterized as a widened, thickened, and raised scar, often associated with pruritus, and typically develops at the site of skin injury [19]. Scar formation represents an efficient evolutionary repair mechanism in human genetics, yet once formed, it is challenging to completely eradicate [5]. Despite this, the precise pathogenesis remains incompletely understood. Research has demonstrated that alterations in miR‐431‐5p expression can influence the metabolic and deposition processes of the extracellular matrix, thereby contributing to the pathological development of HS [16]. Moreover, *ZEB1* exhibits a markedly upregulated trend in HS tissues [18]. Based on these findings, we hypothesized that the miR‐431‐5p/*ZEB1* axis may play a pivotal role in the formation and progression of HS. To validate this hypothesis, we enrolled 100 HS patients in our study and observed a significant reduction in miR‐431‐5p expression within HS tissues, implying that its downregulation might represent a critical factor underlying abnormal scar hyperplasia.

Studies have demonstrated that miR‐431‐5p is significantly downregulated in the synovial fibroblast‐like cells of rheumatoid arthritis patients [20]. Moreover, prior research has indicated that overexpression of miR‐431‐5p can inhibit the proliferation of pancreatic ductal adenocarcinoma cells, induce apoptosis, and modulate the cell cycle [21]. Our findings further elucidated that miR‐431‐5p served as a critical regulatory factor for maintaining normal cellular behavior, with functions including inhibition of cell proliferation and migration and promotion of apoptosis. Reduced miR‐431‐5p disrupted the dynamic equilibrium among cell proliferation, migration, and apoptosis, resulting in excessive proliferation and migration alongside diminished apoptosis, which ultimately contributed to the persistent growth of scar tissue. Additionally, miR‐431‐5p exhibited anti‐inflammatory and anti‐fibrotic effects by downregulating inflammatory cytokines and suppressing *α‐SMA* expression. Consequently, low expression of miR‐431‐5p may induce chronic inflammation and excessive fibrosis, thereby facilitating scar formation.

In normal fibroblasts induced by *TGF‐β1*, *ZEB1* is significantly upregulated, which is accompanied by an increase in *α‐SMA* expression [18]. During liver fibrosis, *ZEB1* has been shown to exhibit an upward trend [22, 23]. Furthermore, *ZEB1* promotes the invasive behavior of lung cancer by stabilizing and depositing collagen mediated by *LOXL2* in the extracellular matrix [24]. In vitro experiments on human cardiac fibroblasts revealed that knocking out *ZEB1* effectively reduces *α‐SMA* expression levels [25]. This study further elucidated that *ZEB1* was a critical downstream target of miR‐431‐5p. Overexpression of *ZEB1* could partially counteract the function of miR‐431‐5p, suggesting a dynamic equilibrium between the two. Under normal physiological conditions, this balance ensured cellular function stability and tissue homeostasis. However, in HS tissues, due to the low expression of miR‐431‐5p and high expression of *ZEB1*, this equilibrium was disrupted, resulting in abnormal cellular behavior and promoting scar formation. In the future, monitoring the expression of miR‐431‐5p and *ZEB1* in patients and dynamically tracking their changes during treatment could facilitate the evaluation of therapeutic efficacy and disease progression. Additionally, regulating these molecules' expression, such as developing drugs or therapies to upregulate miR‐431‐5p or inhibit *ZEB1* activity, may offer novel strategies for personalized medicine and provide effective approaches for treating HS.

In addition, this study had certain limitations that warrant acknowledgment. The sample size was relatively small, and an animal model had not yet been established. Future research will focus on expanding the diversity and quantity of samples more effectively. Simultaneously, we plan to select suitable experimental animals to construct an HS animal model and validate the findings of this study by correlating them with clinical patient data. This approach will facilitate a more comprehensive elucidation of the mechanisms underlying miR‐431‐5p and *ZEB1* in HS, thereby enhancing the universality and reliability of our conclusions.

## 5 Conclusions

In summary, miR‐431‐5p exhibited low expression in HS tissues. miR‐431‐5p negatively regulated *ZEB1* and participated in the progression of HS. Consequently, miR‐431‐5p may serve as a potential therapeutic target for HS, offering new insights into treatment strategies based on this molecular axis.

## Author Contributions

Conceptualization: Suwen Zhang and Junjie Liao; Methodology: Xiaolin Miao, Suwen Zhang; Formal analysis and investigation: Xiaolin Miao, Yaqing Liu, Tonghao Yao and Junjie Liao; Writing – original draft preparation: Xiaolin Miao; Writing – review and editing: Suwen Zhang, Yaqing Liu, Tonghao Yao and Junjie Liao; Resources: Suwen Zhang; Supervision: Suwen Zhang.

## Ethics Statement

This study was approved by the Ethics Committee of Tongde Hospital of Zhejiang Province. Both patients and their family members provided written informed consent, and all experimental procedures were conducted in strict accordance with the guidelines outlined in the Declaration of Helsinki.

## Conflicts of Interest

The authors declare no conflicts of interest.

## Data Availability

All data generated or analyzed during this study is included in this article. Further enquiries can be directed to the corresponding author.
